# Positive and Negative Symptoms of Schizophrenia and Polymorphic Variants of the *TCF4* Gene: Pilot Associative Study

**DOI:** 10.3390/ijms262110507

**Published:** 2025-10-29

**Authors:** Svetlana A. Ivanova, Vladimir V. Tiguntsev, Anastasia S. Boiko, Ekaterina V. Mikhalitskaya, Dmitry A. Petkun, Irina A. Mednova, Olga Yu. Fedorenko, Nikolay A. Skryabin, Elena G. Kornetova, Alexander N. Kornetov, Nikolay A. Bokhan

**Affiliations:** 1 Mental Health Research Institute, Tomsk National Research Medical Center, Russian Academy of Sciences, Tomsk 634014, Russia; cristall2009@live.ru (V.V.T.); anastasya-iv@yandex.ru (A.S.B.); uzen63@mail.ru (E.V.M.); substantia_p@mail.ru (D.A.P.); irinka145@yandex.ru (I.A.M.); f_o_y@mail.ru (O.Y.F.); ekornetova@outlook.com (E.G.K.); alkornetov@gmail.com (A.N.K.); bna909@gmail.com (N.A.B.); 2 Psychiatry, Addictology and Psychotherapy Department, Siberian State Medical University, Tomsk 634050, Russia; 3 Research Institute of Medical Genetics, Tomsk National Research Medical Center of the Russian Academy of Sciences, Tomsk 634050, Russia; skryabinn@mail.ru

**Keywords:** molecular genetics, TCF4, single nucleotide polymorphism, clinical heterogeneity, schizophrenia

## Abstract

The role of the genetic component in the development of schizophrenia and the formation of its clinical heterogeneity has been proven. To conduct a pilot associative analysis between positive and negative schizophrenia symptoms and polymorphic variants of the Transcription Factor 4 (*TCF4*) gene. The study included 373 patients with schizophrenia of Caucasian ethnicity, who underwent a comprehensive clinical examination, and a control group consisted of 194 mentally and somatically healthy individuals. Genotyping of three polymorphic variants of the *TCF4* gene was carried out in the studied samples (rs2958182, rs8766, and rs9636107). Statistical analysis of the results was performed using Statistica for Windows V.12.0. Association analysis in SNPs was conducted using the chi-square criterion and Bonferroni correction. Groups of schizophrenia patients and healthy individuals were compared for selected *TCF4* gene polymorphisms. No statistically significant differences in genotype and allele frequencies were found. The AA genotype and the A allele of the rs2958182 polymorphic variant, as well as the A allele of the rs9636107 polymorphic variant, had an effect predisposing to the predominance of negative symptoms. The TT genotype and the T allele of the rs2958182 polymorphic variant, as well as the G allele of the rs9636107 polymorphic variant, were statistically significantly more common among patients with leading positive symptoms. As a result of the study, associations of the polymorphic variant *TCF4* rs2958182 and *TCF4* rs9636107 with the leading symptoms of schizophrenia were discovered for the first time in Caucasian populations of the Siberian region. The obtained data confirm the contribution of the genetic component to the formation of clinical heterogeneity of schizophrenia and open up prospects for further search for genetic markers in order to prevent an unfavorable outcome of the disease.

## 1. Introduction

Schizophrenia is a chronic progressive disease of a multifactorial nature, leading to disability and social maladjustment in patients. Despite significant efforts in the development of new treatment methods and programs of social support and rehabilitation of patients, schizophrenia remains a socially significant disease [1].

In modern psychiatry, the clinical symptoms of schizophrenia are divided into several domains. The main features of the disease are described through positive, negative symptoms and cognitive deficit (DSM-5, 2013). The mutual overlap of these symptoms forms the clinical polymorphism of schizophrenia. Patients may have both positive symptoms (delusions, hallucinations, and psychomotor agitation) and negative symptoms (abulia, anhedonia, autism, and decreased emotional response), which can affect the prognosis of the disease [2,3]. Positive symptoms are easier to treat and are associated with a more favorable prognosis, in contrast to negative symptoms [4]. The type of schizophrenia course and the age of its manifestation also have prognostic value. An episodic type of course with the presence of remissions, when the symptoms of the disease are smoothed out or disappear completely, and the patient develops criticism of his own condition, is considered more favorable in comparison with a continuous type of course [5]. An early age of disease manifestation (up to 18 years) is considered unfavorable in prognostic terms [6,7].

The Transcription factor 4 (*TCF4*) gene is located in the 18q21 region and encodes transcription factor 4, a protein that recognizes the Ephrussi-box binding site (“E-box”) (‘CANNTG’). This gene is widely expressed in the body and may play an important role in the development of the nervous system [8,9] and embryogenesis [10].

The role of the genetic component in the development of schizophrenia and the formation of its clinical picture has been proven [11,12]. The polymorphic variant *TCF4* rs2958182, as shown in samples in the European [13] and Chinese populations [14], is part of the locus of predisposition to schizophrenia. There are also opposite results showing the absence of the above association [15,16,17]. However, in 2020, a meta-analysis was conducted that confirmed the contribution of *TCF4* rs2958182 to the predisposition to the development of schizophrenia [18].

In 2016, a team of authors led by Chow T.J. found an association of the *TCF4* rs8766 polymorphic variant with an early age of schizophrenia onset [19], but the statistical significance of these results disappeared after adjusting for false positives. Similar work was conducted in 2012 in Norway [20]. Within the framework of this study, a stable association was found between several polymorphic variants of the *TCF4* gene, in particular rs9636107, with an early age of schizophrenia onset, as well as several others, with reduced levels of verbal fluency and executive functions in patients with schizophrenia. In other ethnic populations, the association of polymorphic variants of the *TCF4* gene with schizophrenia has not been studied.

Based on the above, we hypothesized that polymorphic variants of the *TCF4* gene may be involved in the development of leading positive or negative symptoms.

Thus, the aim of our pilot study was to analyze the possible associations between positive and negative schizophrenia symptoms and polymorphic variants of the TCF4 gene.

## 2. Results

The main group of subjects included 373 patients with schizophrenia, and the control group consisted of 194 mentally and somatically healthy individuals. The distribution by gender in the patient and control groups was equal. The sociodemographic and clinical characteristics of the patients, including the duration of the disease, the type of schizophrenia course and the leading symptoms, are presented in Table 1.

The data on the frequency distribution of genotypes (rs2958182, rs8766, rs9636107) were tested for compliance with HWE (Hardy–Weinberg equilibrium) (Table 2). All polymorphic variants were consistent with equilibrium (*p* > 0.05).

The characteristics of polymorphic variants of the *TCF4* gene and the minor allele frequencies are presented in Table 3. Minor allele frequencies in our population of mentally and somatically healthy individuals (the control group) were consistent with those in European populations represented in the 1000 Genomes Project.

In the first stage of the study, groups of schizophrenia patients and healthy individuals were compared for selected *TCF4* gene polymorphisms. No statistically significant differences in genotype and allele frequencies were found (Table 4).

We proceeded from Crow’s dichotomous concept of schizophrenia (positive and negative) for psychopathological estimation [21]. This concept postulates two “pathological aspects” underlying schizophrenia: a positive component (potentially sensitive to antipsychotics) and a negative component (often progressive and associated with a deficit state and poor long-term outcome). Positive symptoms manifest themselves in the form of hallucinations and delusions; negative symptoms include flattening of emotions and avolition. The described symptoms are characterized by either a continuous or episodic course, which, in contrast to the former, is manifested by exacerbations of schizophrenia episodes and then remissions. As the disorder develops, negative symptoms come to the forefront of the clinical picture in a significant proportion of patients. To investigate the involvement of the studied genetic variants in the progression of predominant negative or positive symptoms according to the PANSS survey data, the initial sample of schizophrenia patients was split into two subgroups: a subgroup with predominant negative symptoms and a subgroup of patients with predominant positive symptoms. Comparison of the frequencies of genotypes and alleles of the *TCF4* gene polymorphic variants in groups of patients with different leading symptoms of schizophrenia is presented in Table 5. The AA genotype (χ^2^ = 11.32; *p* = 0.003; OR = 2.77; 95% CI: 1.37–5.59) and the A allele (χ^2^ = 10.06; *p* = 0.002; OR = 1.67; 95% CI: 1.23–2.28) of the rs2958182 polymorphic variant, as well as the A allele of the rs9636107 polymorphic variant (χ^2^ = 6.97; *p* = 0.009) had an effect predisposing to the predominance of negative symptoms, while the TT genotype (OR = 0.59; 95% CI: 0.39–0.89) and the T allele (OR = 0.60; 95% CI: 0.44–0.82) of the polymorphic variant rs2958182, as well as the G allele of the rs9636107 polymorphic variant, were statistically significantly more common among patients with leading positive symptoms.

In addition to the association analysis, binary logistic regression was conducted, with schizophrenia-related symptoms as the dependent variable and sociodemographic, clinical, and genetic factors as independent covariates (Table 6). The analysis revealed that the disease duration and the TT and TA genotypes of the rs2958182 polymorphism all contribute statistically significantly to the development of the schizophrenia leading symptoms. Gender, age, age of manifestation, chlorpromazine equivalent, pharmacological profile of antipsychotics, AA genotype of the rs2958182 polymorphism, and genotypes of the other studied polymorphisms were excluded at different stages of regression formation as nonsignificant covariates.

## 3. Discussion

Despite the unquenchable interest of researchers in the problem of genetic predisposition to schizophrenia, the contribution of polymorphic variants of transcription factor genes to the formation of the clinical picture of schizophrenia has still not been sufficiently studied in the scientific community.

The basic helix–loop–helix transcription factor 4 had been identified as a susceptibility gene associated with schizophrenia by GWAS, but inconsistent results have been found in other studies [14,15].

The *TCF4* gene is associated with many mental disorders and deficiencies, and this gene is suggested as a gene candidate of high risk for SCZ [22].

Two polymorphic variants (rs9960767 and rs2958182) of *TCF4* have been identified as susceptibility loci for SCZ, as confirmed by studies in a European population (16,161 samples) and a Han Chinese population (7680 individuals), respectively [13,14]. However, a number of studies have shown nonsignificant associations between rs2958182 and SCZ in European and Han populations [15,16,17].

In our study, we attempted to replicate the results of genome-wide studies on three gene *TCF4* polymorphisms (rs2958182, rs8766, and rs9636107) and the risk of developing schizophrenia; however, we did not obtain statistically significant differences between our population of patients with schizophrenia and healthy individuals. Our negative results are consistent with a number of replication studies. Thus, Tavakkoly-Bazzaz J et al. [23] genotyped rs8766 in *TCF4* in a set of Iranian case-control samples, including 215 patients and 220 matched healthy controls, and found that rs8766 allele and genotype frequencies were not significantly different between the case and control groups. Four SNPs (rs2958182, rs1261085, rs8766, and rs12966547) showed nonsignificant associations between patients and controls in the Northwest Han Chinese population [18]. The conflicting results may be due to differences in sample sizes, clinical heterogeneity of schizophrenia, ethnic characteristics, geographic distribution, and diagnostic methods in the study populations.

Based on Crowe’s dichotomous concept, we focused on the association analysis of gene polymorphisms with dominant negative or positive symptoms, as measured using the PANSS scale. We found that the AA genotype and the A allele (χ^2^ = 10.06; *p* = 0.002) of the rs2958182 polymorphic variant, as well as the A allele of the rs9636107 polymorphic variant, had an effect predisposing to the predominance of negative symptoms. The TT genotype and the T allele of the rs2958182 polymorphic variant, as well as the G allele of the rs9636107 polymorphic variant, were statistically significantly more common among patients with leading positive symptoms. The study of genetic variability of TCF4 in schizophrenia of the Southern Chinese Han population identified associations with positive and negative symptoms, but these associations were not corrected for multiple testing [24]. Alizadeh F et al. [25] found that the TCF4 level is negatively correlated with the PANSS score. Their results showed that the mRNA level of TCF4 may be associated with SCZ and its psychopathology in an Iranian group of patients with SCZ. Minor alleles of eleven SNPs (rs1261086, rs1261073, rs1261076, rs1942265, rs7241077, rs1893431, rs4800988, rs1261134, rs1788027, rs1660242, and rs1788014) from sequential LD blocks were associated with less negative symptoms in patients with schizophrenia [20]. There was no significant association with positive symptoms. The TCF4 mRNA expression level in blood was significantly increased in psychosis patients compared with controls and positively correlated with positive- and negative-symptom levels [20].

It is interesting that the polymorphic variants affecting negative symptoms are located in the gene’s exon-dense region, where mutations causing Pitt–Hopkins syndrome have been identified [26]. This is a rare hereditary disease characterized by mental retardation, autistic features, severe delay in motor and psychomotor development, and other features [27]. The clinical picture of children with autistic features and developmental delay is partly similar to that of schizophrenia with leading negative symptoms and severe neurocognitive deficit; neuroimaging studies show an increased volume of the cerebral ventricles in children with Pitt–Hopkins syndrome [28], so the contribution of polymorphic variants of the *TCF4* gene to the formation of independent clinical, cognitive, and neuroimaging phenotypes appears systemic.

In both genome-wide and replication studies, as well as in our study, all polymorphic variants of the *TCF4* gene are localized within an intron. Possible molecular mechanisms by which intronic variations may play an important regulatory role include linkage with unknown causal DNA or constitute potential regulatory elements for distal genes, location in trans-splicing elements, alterations in transcription factor binding or enhancer activity, and effect on posttranslational histone modifications. Further elucidation of the causal relationship between polymorphic variants, *TCF4* gene regulation, and clinical heterogeneity, progression, and outcome is important for understanding the pathogenesis of schizophrenia.

Limitations of the study: Our research has several limitations. It is a pilot study that conducted a small-scale preliminary assessment to test feasibility, refine the research design, and attract the attention of other researchers to this topic. The main limitation is the small size of the subgroups, which can reduce statistical power and increase the risk of Type I and II errors. Our findings are preliminary and therefore require replication of the obtained data for other cohorts. Symptoms were assessed during the diagnostic interview and may therefore reflect a treatment effect, as the patients studied were receiving pharmacological treatment with antipsychotic medications; thus, we cannot distinguish the association of genotype with the underlying symptom profile from its association with treatment response. Our investigation is a cross-sectional study, and we do not have complete details on clinical subgroups of the sample (e.g., presence of comorbid cognitive impairment) that would lay the foundation for deep stratified analysis. The next limitation is the number of polymorphisms studied relative to the complexity of *TCF4.* Furthermore, we do not analyze gene–environment interaction that can influence the clinical characteristics of patients. The fact that only Caucasians were included in this study limits its generalizability, although we attempted to achieve ethnic homogeneity in the sample.

## 4. Materials and Methods

### 4.1. Patients and Healthy Volunteers

This cross-sectional, case-control study included 373 patients with schizophrenia from the Siberian Federal District (Russia). Patients were recruited from the clinics of the Mental Health Research Institute, Tomsk National Research Medical Center, the Tomsk Clinical Psychiatric Hospital, the Hospital of the Siberian State Medical University, and the Kemerovo Regional Clinical Psychiatric Hospital.

The patient group included 180 women and 193 men of Caucasian ethnicity. All patients included in the study underwent inpatient treatment, signed voluntary informed consent and received therapy with antipsychotic drugs in the average therapeutic doses recommended by the manufacturer.

Inclusion criteria: (1) Age 18–65 years; (2) at least 1 year of follow-up; (3) belonging to the Caucasian ethnicity; (4) verified diagnosis of schizophrenia according to ICD-10 criteria F20.0, paranoid schizophrenia; (5) consent to participate in the study.

Exclusion criteria: (1) Non-Caucasian physical appearance (e.g., Mongoloid, Buryats, or Khakassians), organic, neurological, severe somatic diseases leading to organ failure; (2) the presence of concomitant addictive or other mental disorders; (3) refusal to participate in the study.

All subjects completed the “Basic card of sociodemographic and clinical-dynamic features for patients with schizophrenia”, which we had previously tested in clinical studies.

The control group consisted of 194 mentally and somatically healthy individuals (92 males and 102 females) without chronic diseases and signs of acute infectious diseases at the time of examination.

### 4.2. Psychometric Assessment

The severity of the mental state was verified using the Positive and Negative Syndrome Scale (PANSS) [29] in the adapted Russian version.

The continuous or episodic course of the disorder was concluded on the basis of the fifth character of ICD-10.

Data were gathered regarding baseline antipsychotic therapy and any concomitant treatments at the time of the examination and over the preceding six months (medicines and doses administered and duration of current medication use). For dose standardization, the daily dose of a chlorpromazine equivalent (CPZeq) was used.

### 4.3. Molecular Genetic Analysis

DNA from venous peripheral blood was isolated using the standard phenol–chloroform method. Real-time PCR was performed using primers by DNA-Synthesis (Russia), and BioMaster UDG HS-qPCR Lo-ROX (2x) (Biolabmix, Novosibirsk, Russia), which includes highly processive recombinant HS-Taq DNA polymerase; Deoxynucleoside triphosphate mixture; PCR buffer; mg^2+^; ROX dye on the amplifier QuantStudio™ 5 Real-Time PCR System (Applied Biosystems, Waltham, MA, USA). The equipment is located at the core facility of Medical Genomics, Tomsk National Research Medical Center, Russian Academy of Sciences. PCR was performed according to the standard protocol recommended by the manufacturer in duplicates, with negative controls included. Samples with call rates less than 90% were excluded from further analysis.

The polymorphic variants of the rs2958182, rs8766, and rs9636107 were selected for genotyping. These gene polymorphisms were chosen for the following reasons:

- Minor allele frequency of at least 5%;
- Availability of information on previous studies of this polymorphism;
- Marker localization.

We calculated a formal power analysis for each SNP–phenotype test to pre-empt concerns about modest effect sizes typical for TCF4 loci. Post hoc power analysis revealed 97% power (α = 0.01, df = 2) to detect the observed effect size (Cohen’s *w* = 0.25) for the associated SNP (for rs2958182 and rs9636107), given the sample size of 360 and 363, respectively. For rs8766, post hoc power analysis revealed 93% power (α = 0.01, df = 2) to detect the observed effect size (Cohen’s *w* = 0.25), given the sample size of 306.

### 4.4. Statistical Analysis

Statistical analysis was performed using Statistica for Windows V.12.0 software (StatSoft, Tulsa, OK, USA). The distribution agreement with the normal law was tested using the Shapiro–Wilk criterion. The obtained data did not obey the normal distribution law, so they are presented as a median with an interquartile range—Me [Q1; Q3]. Qualitative data are presented as frequency indicators in absolute and relative units—*n* (%). The modified chi-square test (χ^2^) was used to test the correspondence of the genotype and allele frequency distribution of the studied gene to the Hardy–Weinberg distribution (HWD). When comparing qualitative data, Pearson’s χ^2^ was used, including taking into account the Yates correction, and Fisher’s exact test (if one or more of the study groups had fewer than five people). Association analysis in SNPs was conducted using the chi-square criterion and Bonferroni correction. Corrected statistical significance *p* = 0.05/3 = 0.017. After the false discovery rate (FDR = 0.033) correction (Q = 5%), all statistically significant differences remained too. Comparison of quantitative data was performed using the Kruskal–Wallis criterion (H). The odds ratio (OR) with the calculation of the 95% confidence interval was used as a quantitative measure of the degree of association of the genetic marker with the clinical indicator. The threshold level of statistical significance was *p* = 0.05.

## 5. Conclusions

Thus, for the first time, we have discovered associations of the polymorphic variant *TCF4* rs2958182 and *TCF4* rs9636107 with the leading symptoms of schizophrenia in Caucasian patients of the Siberian region. Further study of polymorphic variants of the *TCF4* gene opens up prospects for further search for genetic markers of schizophrenia and a deeper understanding of the nature of psychotic disorders.

## Figures and Tables

**Table 1:** Sociodemographic and clinical characteristics of the examined patients.

Parameter	Value
Sample size, *n*	373
Sex, *n* (%)	Men: 193 (51.7%)
	Women: 180 (48.3%)
Age, years Me (Q1; Q3)	38 (33; 48)
Age of manifestation, years Me (Q1; Q3)	24 (20; 30)
Duration of disease, years Me (Q1; Q3)	14 (6; 22)
PANSS Me (Q1; Q3)	Total score: 104 (94; 113)
	Positive symptoms: 23 (19; 27)
	Negative symptoms: 26 (23; 29)
	General psychological symptoms: 54 (49; 60)
Leading symptoms, *n* (%)	Positive: 184 (49.3%)
	Negative: 189 (50.7%)
Type of course, *n* (%)	Episodic: 127 (34.0%)
	Continuous: 186 (50.7%)
	Observation period less than 1 year: 22 (5.9%)
	Unknown 38 (10.2%)
Duration of basic therapy, years Me (Q1; Q3)	8 (2; 16)
Chlorpromazine equivalent, mg Me (Q1; Q3)	425 (250; 763)
Pharmacological profile of antipsychotics, *n* (%)	Conventional: 206 (55.2)
	Atypical: 167 (44.8)

Notes. Me (Q1; Q3): median and quartiles (first and third).

**Table 2:** Hardy–Weinberg equilibrium in a group of patients with schizophrenia.

Polymorphic Variant			Allele Frequencies, %	Missing Values, %	χ^2^	*p*
rs2958182	alleles	T	65.6	2.7	<0.001	0.992
		A	34.4			
rs8766	alleles	T	58.2	1.3	1.361	0.243
		C	41.8			
rs9636107	alleles	G	52.9	1.9	0.127	0.362
		A	47.1			

Notes. *p* HWE: the *p*-value of the Hardy–Weinberg equilibrium test.

**Table 3:** Characteristics of polymorphic variants of the TCF4 gene and minor allele frequency.

SNP	Chromosome, Position	Alleles	Type	MAF (Study)	MAF (Global)	MAF (Europe)
rs9636107	Chr.18:55532886	A/G	Intron Variant Transition substitution	0.476	0.40	0.49
rs2958182	Chr.18:55481790	A/T	Intron Variant Transversion substitution	0.335	0.23	0.33
rs8766	Chr.18:55228300	C/T	Intron Variant Transition substitution	0.422	0.34	0.42

Note: MAF (study): Our observed MAF values for healthy individuals; MAF from the Ensemble database based on the 1000 Genomes Project.

**Table 4:** Comparison of genotype and allele frequencies of polymorphic variants of the *TCF4* gene in groups of patients with schizophrenia and healthy individuals.

Polymorphic Variant			Healthy Individuals, *n* (%)	Patients with Schizophrenia, *n* (%)	OR		χ^2^/F	*p*
					OR	95% CI		
rs2958182	genotypes	TT	77 (41)	156 (43)	0.92	(0.64–1.32)	2.79	0.25
		TA	96 (51.1)	164 (45.2)	1.27	(0.89–1.80)		
		AA	15 (7.9)	43 (11.8)	0.64	(0.35–1.19)		
	alleles	T	250 (66.5)	476 (65.6)	1.04	(0.80–1.36)	0.06	0.81
		A	126 (33.5)	250 (34.4)	0.96	(0.74–1.25)		
rs8766	genotypes	TT	63 (33.7)	119 (32.3)	1.06	(0.73–1.55)	0.71	0.7
		CT	90 (48.1)	190 (51.6)	0.87	(0.61–1.24)		
		CC	34 (18.2)	59 (16)	1.16	(0.73–1.85)		
	alleles	T	216 (57.8)	428 (58.2)	0.98	(0.77–1.27)	0.004	0.95
		C	158 (42.2)	308 (41.8)	1.02	(0.79–1.31)		
rs9636107	genotypes	GG	46 (24.5)	104 (28.4)	0.82	(0.55–1.22)	2.39	0.31
		GA	105 (55.9)	179 (48.9)	1.32	(0.93–1.88)		
		AA	37 (19.7)	83 (22.7)	0.84	(0.54–1.29)		
	alleles	G	197 (52.4)	387 (52.9)	0.98	(0.77–1.26)	0.007	0.93
		A	179 (47.6)	345 (47.1)	1.02	(0.80–1.31)		

Note: OR—odds ratio; CI—confidence interval.

**Table 5:** Comparison of the frequencies of genotypes and alleles of the *TCF4* gene polymorphic variants in groups of patients with different leading symptoms of schizophrenia.

Polymorphic Variant			Patients with Negative Symptoms, *n* (%)	Patients with Positive Symptoms, *n* (%)	OR		χ^2^/F	*p*
					OR	95% CI		
rs2958182	genotypes	TT	67 (36.4)	87 (49.4)	0.59	(0.39–0.89)	11.32	0.003 *
		TA	86 (46.7)	77 (43.8)	1.13	(0.75–1.71)		
		AA	31 (16.8)	12 (6.8)	2.77	(1.37–5.59)		
	alleles	T	220 (59.8)	251 (71.3)	0.60	(0.44–0.82)	10.06	0.002 *
		A	148 (40.2)	101 (28.7)	1.67	(1.23–2.28)		
rs8766	genotypes	TT	63 (33.9)	55 (30.7)	1.16	(0.74–1.79)	0.53	0.78
		CT	93 (50)	96 (53.6)	0.87	(0.57–1.30)		
		CC	30 (16.1)	28 (15.6)	1.04	(0.59–1.82)		
	alleles	T	219 (58.9)	206 (57.5)	1.06	(0.79–1.42)	0.08	0.77
		C	153 (41.1)	152 (42.5)	0.95	(0.71–1.27)		
rs9636107	genotypes	GG	44 (23.7)	59 (33.3)	0.62	(0.39–0.98)	7.33	0.026
		GA	90 (48.4)	87 (49.2)	1.01	(0.67–1.53)		
		AA	52 (28)	31 (17.5)	1.83	(1.11–3.02)		
	alleles	G	178 (47.9)	205 (57.9)	0.67	(0.50–0.90)	6.97	0.009 *
		A	194 (52.1)	149 (42.1)	1.50	(1.12–2.01)		

Note: *—statistically significant differences; OR—odds ratio; CI—confidence interval.

**Table 6:** Determinants of leading symptoms in binary regression analysis, with gender, age, age of manifestation, disease duration, chlorpromazine equivalent, pharmacological profile of antipsychotics, and investigated SNPs as independent variables.

Variables	B	*p*-Value	Exp (B)	95% CI
Disease duration	−0.071	0.000	0.932	0.909–0.955
TT rs2958182	1.202	0.006	3.326	1.420–7.794
TA rs2958182	0.998	0.022	2.713	1.157–6.362
Constant	0.009	0.982	1.009	

Notes: B—logistic regression equation coefficient, Exp (B)—odds ratio.

## Data Availability

The datasets generated for this study will not be made publicly available, but they are available upon reasonable request from Svetlana A. Ivanova (ivanovaniipz@gmail.com), following approval of the Board of Directors of the MHRI, in line with local guidelines and regulations.

## Abbreviations

PANSS	Positive and Negative Syndrome Scale
TCF4	Transcription factor 4
